# Antifungal Effect of Copper Nanoparticles against *Fusarium kuroshium*, an Obligate Symbiont of *Euwallacea kuroshio* Ambrosia Beetle

**DOI:** 10.3390/jof8040347

**Published:** 2022-03-27

**Authors:** Enrique Ibarra-Laclette, Jazmín Blaz, Claudia-Anahí Pérez-Torres, Emanuel Villafán, Araceli Lamelas, Greta Rosas-Saito, Luis Arturo Ibarra-Juárez, Clemente de Jesús García-Ávila, Arturo Isaías Martínez-Enriquez, Nicolaza Pariona

**Affiliations:** 1 Instituto de Ecología, A.C. (INECOL), Red de Estudios Moleculares Avanzados (REMAV), Xalapa 91073, Veracruz, Mexico; jazmin-itzel.blaz-sanchez@u-psud.fr (J.B.); claudia.perez@inecol.mx (C.-A.P.-T.); emanuel.villafan@inecol.mx (E.V.); araceli.lamelas2@gmail.com (A.L.); greta.rosas@inecol.mx (G.R.-S.); luis.ibarra@inecol.mx (L.A.I.-J.); 2 Centre National de la Recherche Scientifique (CNRS), Unité d’Ecologie Systématique et Evolution, Diversity, Ecology and Evolution of Microbes Team (DEEM), Université Paris-Saclay, AgroParisTech, 91405 Orsay, France; 3 Investigador por México-CONACYT en el Instituto de Ecología, A.C. (INECOL), Xalapa 91073, Veracruz, Mexico; 4 Dirección General de Sanidad Vegetal-Centro Nacional de Referencia Fitosanitaria (DGSV-CNRF), Tecamac 55740, Mexico; clemente.garcia@senasica.gob.mx; 5 Centro de Investigación y de Estudios Avanzados del IPN Unidad Saltillo, Coahuila 25900, Mexico; arturo.martinez@cinvestav.edu.mx

**Keywords:** nanofungicide, antifungal activity, ambrosial complex

## Abstract

Copper nanoparticles (Cu-NPs) have shown great antifungal activity against phytopathogenic fungi, making them a promising and affordable alternative to conventional fungicides. In this study, we evaluated the antifungal activity of Cu-NPs against *Fusarium kuroshium*, the causal agent of *Fusarium* dieback, and this might be the first study to do so. The Cu-NPs (at different concentrations) inhibited more than 80% of *F. kuroshium* growth and were even more efficient than a commercial fungicide used as a positive control (cupric hydroxide). Electron microscopy studies revealed dramatic damage caused by Cu-NPs, mainly in the hyphae surface and in the characteristic form of macroconidia. This damage was visible only 3 days post inoculation with used treatments. At a molecular level, the RNA-seq study suggested that this growth inhibition and colony morphology changes are a result of a reduced ergosterol biosynthesis caused by free cytosolic copper ions. Furthermore, transcriptional responses also revealed that the low- and high-affinity copper transporter modulation and the endosomal sorting complex required for transport (ESCRT) are only a few of the distinct detoxification mechanisms that, in its conjunction, *F. kuroshium* uses to counteract the toxicity caused by the reduced copper ion.

## 1. Introduction

The applications of nanotechnology have significantly increased over the last few years. Currently, different nanomaterials are being used in agriculture, creating a new field known as nanoagriculture. Various nanomaterials with antimicrobial activity have been tested for the control of infectious diseases, such as Ag nanoparticles (NPs) [1], Au-NPs [2], TiO_2_-NPs [3], and ZnO-NPs [4]. Copper-based nanoparticles have drawn particular interest due to their low cost, excellent antimicrobial properties, and minimal environmental impact when used correctly (low concentrations with highly efficient modifications/formulations) [5]. For centuries, copper salts have been used for disease control [6]. One of their main advantages is that pathogens do not develop resistance to them, as occurs with most antibiotics [7]. However, due to their high dissolution in water, the cumulative dosages may be toxic to fish and other organisms [8].

Due to their unique physicochemical features, copper-based NPs have shown high antifungal properties against a broad spectrum of fungi species, including *Phoma destructive*, *Curvularia lunata*, *Alternaria alternate*, *Fusarium oxysporum*, *Saccharomyces cerevisiae*, among others [9,10]. Previous studies [10,11,12,13,14] have demonstrated that copper NPs antifungal activity depends on their shape, size, and concentration, which could vary depending on the fungal species. Previously, we evaluated the antifungal activity of five Cu/Cu_x_O-NPs with different phase compositions and sizes, using a *Fusarium oxysporum* strain as a study case. The results showed that with a low concentration (0.25 mg/mL) of Cu/Cu_x_O-NPs, with a high proportion of Cu_2_O phase and relatively small size particles, more than 90% of fungal growth was inhibited. Meanwhile, copper salts reached only 5% growth inhibition [11]. Differences were also observed in antifungal activity of Cu-NPs even against species belonging to the same genus (e.g., *Fusarium* sp. AF-6, AF-8, *F. oxysporum,* and *F. solani*).

*Fusarium kuroshium* [15] is a member of the Ambrosia *Fusarium* Clade (AFC) [16,17] and is recognized as one of the symbionts of the Asian Kuroshio shot hole borer (*Euwallacea kuroshio* Gomez and Huler. Since its introduction into the United States of America, this pest has spread from Southern California’s west coast to Northeastern Mexico [18]. The fungus–beetle complex is responsible for causing significant damage to several tree species distributed in urban, natural, agricultural, and riparian areas [17,19,20,21,22,23]. As a control strategy, fungicides from the azole family are commonly used even when they are inefficient. These chemicals can negatively impact ecological interactions and the environment [24]. Hence, it is necessary to find alternatives.

For the first time and based on the framework mentioned above, in this study, we describe the antifungal activity of Cu-NPs exerted against *Fusarium kuroshium* [15]. We analyzed the fungal morphological (growth and development) and molecular response in the presence of Cu-NPs, combining RNA-seq methodology and field emission scanning electron microscopy (FE-SEM).

## 2. Materials and Methods

### 2.1. Source of Fungal Symbionts of Ambrosia Beetles, Media, and Culture Conditions

Under strict biosecurity conditions, all in-vitro assays were carried out in the mycology laboratory at ‘Centro Nacional de Referencia Fitosanitaria (CNRF)’. CNRF is a Mexican institution belonging to ‘Dirección General de Sanidad Vegetal (DGSV)’ and ‘Servicio Nacional de Sanidad, Inocuidad y Calidad Agroalimentaria (SENASICA)’, both dependencies of ‘Secretaría de Agricultura y Desarrollo Rural (SADER)’. The strain HFEW-16-IV-019 of *Fusarium kuroshium* species was used in the present study [19,25,26]. This strain was isolated from the Kuroshio shot hole borer (KSHB), collected in Tijuana, B.C., Mexico, and stored in 25% glycerol at −80 °C [18]. Conidia from *F. kuroshium* were propagated on potato dextrose agar (PDA) (Sigma-Aldrich, St. Louis, MO, USA). Plates were incubated for 5–7 days at 28 °C in darkness, and fungal spores were collected by gently shaking the plate with 3–5 mL of sterile water at room temperature. After the conidia were washed twice with sterile water, they were collected and stored in an aqueous solution (at 5 × 10^6^ colony forming unit (CFU)/mL) and used on the antifungal activity assay.

### 2.2. In Vitro Antifungal Activity Assay

As recently reported, the Cu-NPs used for the in vitro assays were synthesized [27]. These Cu-NPs are faceted particles of 200 nm in size, coated with citrate groups, water dispersible, and stable in the open atmosphere. The commercial fungicide product (Cupravit^®^ Hidro, Bayer de México, CDMX, México) containing the active ingredient cupric hydroxide was used as the positive control and reference of antifungal activity. Sterile distilled water was used as a solvent to prepare both the Cu-NPs suspension and the cupric hydroxide solution. The Cu-NPs suspension was sonicated for 30 min to ensure good dispersion of NPs in the PDA culture medium.

The antifungal activity of Cu-NPs against *F. kuroshium* was evaluated using the poisoned food method [28]. Briefly, PDA was mixed with different amounts of Cu-NPs to obtain the following final concentrations: 0.1, 0.25, 0.5, 0.75, and 1.0 mg/mL. Cupric hydroxide was used at the same concentrations as Cu-NPs, and non-amended media were used as control. Spore suspensions (1 × 10^6^ CFU/mL) were inoculated at the center of each PDA plate and incubated in darkness at 28 °C for six days. All treatments were carried out in triplicate. Colony diameters were measured three and six days after inoculation (dai). The percentage of growth inhibition was calculated by measuring the average area of the fungal colonies in the treatments and compared to the negative control.

### 2.3. Analysis of Fungal Morphology through FE-SEM

Six-day-old fungal from treatment and control cultures were used to determine mycelial radial growth and morphology. Mycelial discs of 10 mm diameter were cut, fixed, and processed as previously described [27,29]. The images were collected using an FE-SEM FEI Quanta 250-FEG (Brno, Czech Republic).

### 2.4. RNA Extraction

Three and six dai mycelium were collected from the Cu-NPs treatments (0.5, 0.75, and 1.0 mg/mL) and control. Samples were immediately frozen in liquid nitrogen and stored at −80 °C for posterior extraction. Total RNA was isolated from 200 mg of pulverized mycelia using Norgen RNA Purification Kit (Nor-gen Biotek Corporation, Thorold, Canada). RNA was quantified using a NanoDrop 2000 c spectrophotometer (Thermo Scientific, Thermo Fisher Scientific, Waltham, MA, USA) and assessed for purity by UV absorbance measurements at 260 and 280 nm. Total RNA integrity was confirmed by capillary electrophoresis using an Agilent 2100 Bioanalyzer (Agilent Technologies, Santa Clara, CA, USA).

### 2.5. RNA-seq Analysis: cDNA Library Preparation and Sequencing

cDNA libraries were prepared by the Massive Sequencing Unit of the Ecology Institute (INECOL, Xalapa, Ver., Mexico) using the TruSeq RNA Sample Preparation Kit (Illumina, San Diego, CA, USA) following the manufacturer’s instructions. A total of 24 samples consisting of three biological replicates of Cu-NPs treatments 0.5, 0.75, and 1 mg/mL and negative control collected at 3 and 6 dai were sequenced. All samples were sequenced together on a single flow cell (High Output Kit v2.5; 300 Cycles) using the NextSeq500 platform (Illumina, San Diego, CA, USA). Paired-end reads (2 × 150 bp) were generated, and index codes were used to identify each sample independently. The RNA-seq data were deposited in the Short Read Archive (SRA) database of the National Center for Biotechnology Information (NCBI). Accession numbers were placed at the end of the manuscript in the data availability statement section.

### 2.6. Data Processing

The resulting raw paired-end reads from the sequencing process were cleaned using Trimmomatic v0.38 [30] to use only high-quality sequences. Reads alignment to the reference genome (*Fusarium kuroshium*; [25,26]) and transcript abundance estimation were performed using Bowtie2 v2.3.5.1 [31] and RNA-Seq by Expectation-Maximization (RSEM) v1.3.1 [32] software packages, respectively. The transcript abundance matrix created contains each of *F. kuroshium* genes (rows) and the expected count (EC) values calculated for each sampling point (3 and 6 dai) at the different concentrations of Cu-NPs employed (0, 0.5, 0.75, and 1 mg/mL; all represented in the corresponding columns). The EC values represent the expression levels and are calculated by the maximum likelihood estimation approach and posterior mean estimates with 95% credibility intervals. RSEM uses these EC values to calculate transcripts per million (TPM) and fragments per kilobase per million mapped reads (FPKM) values. It has been reported that TPM values are highly consistent among samples [33]. These values were used to perform principal component analysis to detect the significant sources of variance underlying the selected sampling points and the Cu-NPs treatments. The DESeq2 v1.2.4.0 R/Bioconductor package performed a differential expression analysis, using a negative binomial model to perform pairwise Wald tests, and the Benjamini–Hochberg method to perform multiple testing [34]. A log_2_ fold change (FC) value ± 1.0 and an adjusted *p* value of ≤ 0.05 were the criteria for identifying differentially expressed genes (DEGs) across treatments.

Considering that gene models predicted in the *F. kuroshium* genome lack annotation [29], its homologs were identified by BLAST searches. Only the best hit in unidirectional pairwise comparisons was considered (*F. kuroshium* versus some other available *Fusarium* species: *F. vanettenii* 77-13-4, *F. graminearum* PH-1, *F. pseudograminearum* CS3096, *F. verticillioides* 7600, *F. fujikuroi* IMI 58289, and *F. oxysporum* NRRL 32931). *Neurospora crassa* OR74A and *Saccharomyces cerevisiae* S288C were also included as outgroups. The names of species mentioned and those used as references are accompanied by the strain identifier (e.g., 77-13-4). The latest versions of these reference genomes, all available in the GenBank database (https://www.ncbi.nlm.nih.gov/; accessed on 17 February 2022), were those used in this study. Gene Ontology (GO) terms [35], eukaryotic orthologous group (KOG), the Enzyme Commission (EC) numbers [36], and Kyoto Encyclopedia of Genes and Genomes (KEGG) pathways [37] were inherited to each *F. kuroshium* gene. InterProScan [38,39] was used for this purpose. The g: Profiler web tool (http://biit.cs.ut.ee/gprofiler/; accessed on 15 February 2022; [40]) was used to identify the enriched functional categories (GO terms) and deep-represented metabolic pathways (KEGG) by genes that respond to the Cu-NPs treatments, significantly changing their transcription level (differentially expressed genes). Finally, GO and KEGG enrichment analysis of the identified DEGs was performed by g: Profiler web tool (http://biit.cs.ut.ee/gprofiler/; accessed on 15 February 2022) using the hypergeometric distribution adjusted by set count sizes (SCS) for multiple hypothesis correction [40]. Based on the method mentioned above (g: SCS), *p*-adjusted values ≤ 0.05 were used as a threshold after performing multiple correction tests.

## 3. Results

### 3.1. Antifungal Activity of Cu-NPs on Mycelial Growth

**Figure 1:**
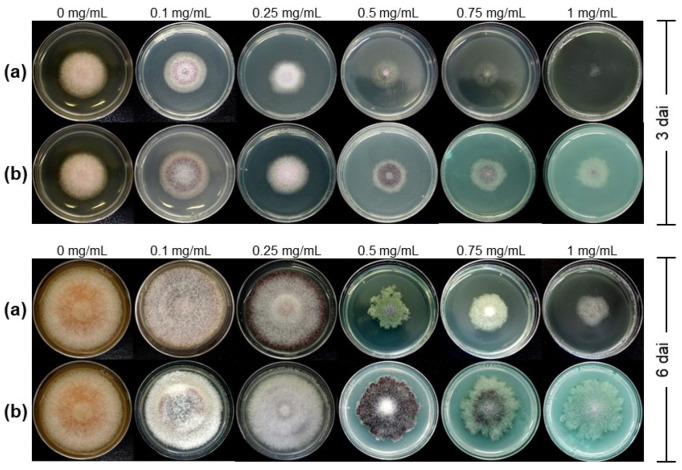
*F. kuroshium* mycelial growth inhibition assays. The colony morphology of *F. kuroshium* wild-type strains grown on plates with PDA culture medium supplemented with different concentrations (0 mg/mL (control), 0.1, 0.25, 0.5, 0.75, and 1 mg/mL) of (**a**) Cu-NPs and (**b**) cupric hydroxide at 3 and 6 days after inoculation (dai).

Both treatments, Cu-NPs and cupric hydroxide, were found to inhibit mycelial growth in a dose-dependent manner. As seen in Figure 1, Cu-NPs had more antifungal activity than cupric hydroxide. Figure 1 shows the radial mycelial growth of *F. kuroshium* exposed to different Cu-NPs and cupric hydroxide concentrations in both sampling points (3 and 6 dai).

At three dai, changes in colony pigmentation and mycelial growth inhibition started to be visible in both treatments (Cu-NPs and cupric hydroxide, respectively). However, colony morphology and percentage of mycelial radial growth inhibition were more evident at 6 dai (Figure 1). At this late sampling time, the *F. kuroshium* colony showed a cotton-like texture and pale orange pigmentation in the negative control (plates with PDA culture medium). In the presence of 0.1 and 0.25 mg/mL Cu-NPs, the color of the colony became white and dark cherry color, and the pigment disappeared when the concentration of Cu-NPs increased from 0.5 to 1 mg/mL. Changes in the colony morphology were also observed (irregular growth), being significant at 0.5 mg/mL.

Regarding the cupric hydroxide treatments (positive control), colony pigmentation changes were also observed from the lowest concentrations (0.1 and 0.25 mg/mL). Still, it turned dark purple g at 0.5 mg/mL (Figure 1).

**Figure 2:**
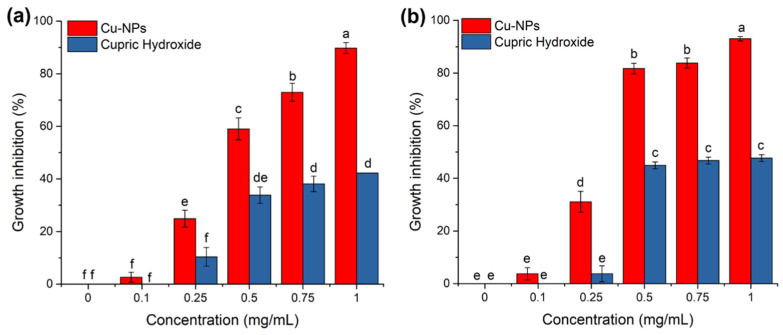
The mycelial radial growth inhibition percentage from F. kuroshium was quantified at (**a**) 3 and (**b**) 6 dai in both treatments, Cu-NPs, and cupric hydroxide, respectively. A one-way ANOVA with a Tukey’s test was used to determine significance across all the treatments. Different letters on top of the bars indicate significant differences (*p* ≤ 0.01). Error bars represent the standard error (*n* = 3).

Additionally, the mycelial radial growth inhibition percentage was quantified (Figure 2). For both treatments (Cu-NPs and cupric hydroxide), growth inhibition became evident at six dai for 0.5, 0.75, and 1 mg/mL concentrations. The mycelial radial growth percentages resulted as higher for Cu-NPs than for cupric hydroxide. As seen in Figure 2, at 0.5 and 0.75 mg/mL of Cu-NPs, ~80% of the fungal growth was inhibited, while at the highest concentration (1 mg/mL), more than 90% inhibition was reached. These growth inhibition percentages were even higher than those observed for the cupric hydroxide treatments, with only 46% inhibition at 0.5 mg/mL and no increase at higher concentrations. As mentioned above, these results revealed that the Cu-NPs treatments at concentrations as low as 0.5 mg/mL might inhibit the growth of *F. kuroshium*, and this treatment seems to perform better than the commercial products available, such as cupric hydroxide, here used as a positive control.

### 3.2. Analysis of Fungal Morphology through FE-SEM

**Figure 3:**
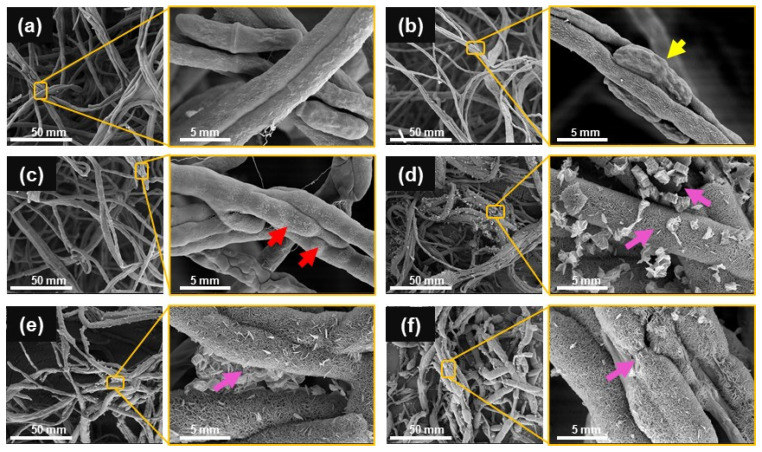
SEM micrographs of *F. kuroshium* hyphae after growing 6 days in PDA culture medium supplemented with different concentrations of Cu-NPs: (**a**) 0 (control), (**b**) 0.1, (**c**) 0.25, (**d**) 0.5, (**e**) 0.75, and (**f**) 1 mg/mL. The Cu-NPs treatments at concentrations as low as 0.1 mg/mL provoked changes in the hyphae morphology, ranging from an apparent loss of turgor to a loss of cell wall integrity. At 0.25 mg/mL, peeling hyphae (red arrow) indicated the loss of cell wall integrity. At concentrations of 0.5 mg/mL or greater, the hyphae cell wall showed higher porosity and leakage of the cytoplasmic contents (pink arrows). The yellow arrow indicates the morphological changes observed in the macroconidia, only found in the control and the 0.1 mg/mL treatment.

FE-SEM micrographs were used to study the structural changes of the fungal hyphae after the treatment by Cu-NPs. In the supplemented control, healthy hyphae exhibited a tubular morphology with a smooth surface and the characteristic formation of fusiform-clavate macroconidia (Figure 3a). In contrast, *F. kuroshium* growing on Cu-NPs treatments showed multiple alterations in the hyphae and macroconidia morphology (Figure 3b–f). At 0.1 mg/mL (Figure 3b), both the hyphae and the macroconidia showed morphological distortion. A reduction in hypha thickness, irregular shrinkages, and peanut shape were observed (yellow arrow). For the 0.25 mg/mL Cu-NPs treatment, there was no production of macroconidia; in addition, hyphae lost their smoothness and exhibited peeling (see red arrows in Figure 3c). At 1 mg/mL of Cu-NPs, *F. kuroshium* hyphae were swollen, deformed, fractured, and broken (pink arrows), leading to the outflow of intracellular components (Figure 3d–f).

### 3.3. Differential Gene Expression of F. kuroshium in Response to Cu-NPs Treatments

A total of 481,775,061 high-quality (HQ) paired-end reads were obtained from the 24 RNA-seq sequenced libraries (around 20 million reads per library on average; Table S1). These HQ reads were mapped against the published *F. kuroshium* genome [25]. From the total of *F. kuroshium* predicted protein-coding genes (13,777), 97.39% were annotated based on *Fusarium vanettenii* (equivalent: *F. solani* f. sp. *pisi*) homologs proteins (Table S2). Homologs proteins were also detected for *Fusarium graminearum* (92.04%), *Fusarium pseudograminearum* (92.80%), *Fusarium verticillioides* (94.27%), *Fusarium fujikuroi* (94.56%), *Fusarium oxysporum* (94.90%), *Saccharomyces cerevisiae* (50.07%), *Neurospora crassa* (80.81%) (Table S2; see Methods for details). The results mentioned above show that, as expected, the amount of homologs proteins identified during the annotation process (homology-based inference) increased as the species they were compared against were phylogenetically more closely related (details in [16]). Tables S3–S5.

**Figure 4:**
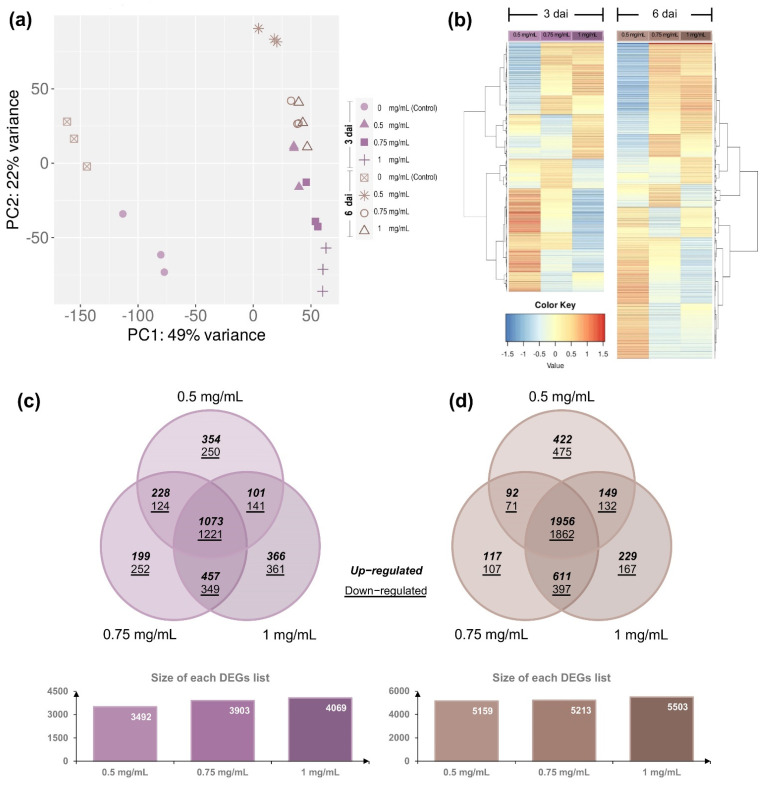
Expression profiles of *Fusarium kuroshium* Differentially Expressed Genes (DEGs) in response to Cu-NPs. (**a**) Principal component analysis (PCA) plot displaying all 24 RNA-seq sequenced libraries used in the presented study, the three independent replicates of the distinct concentrations of Cu-NPs used (0 (control), 0.5, 0.75 and 1 mg/mL) and evaluated at 3 and 6 days after inoculation (dai). PCA was performed using the transcripts per million (TPM) values. (**b**) Heatmaps of the average linkage hierarchical clustering based on the correlation distance measurements. Log_2_FC values (±1) that resulted in significance (adjusted *p* value of ≤ 0.05) were used to represent the lists of DEGs obtained from both the 3 and 6 dai. DEGs lists were generated from pairwise comparisons in which each of the Cu-NPs treatments (0.5, 0.75, and 1 mg/mL) were compared against the control sample (0 mg/mL). The Venn diagram represents the shared amount of up- and downregulated genes in each Cu-NPs treatment at the two sampling points evaluated, 3 dai (**c**) and six dai (**d**).

The principal component analysis (PCA) using the estimated TPM values (Table S6; see Methods for more detail) was conducted to determine the differential expression and to detect the major sources of variance underlying the sampling points (3 and 6 dai) and the Cu-NPs concentrations (0, 0.5, 0.75, and 1 mg/mL). The two-dimensional PC plot in which the first two principal components (PC1 and PC2) were included was the one that best illustrated the variance, with explanatory values of 49% (PC1) and 22% (PC2), respectively (Figure 4a). Since all libraries were independently included in the analysis, the PCA plot indicates that not only the employed biological replicates have high reproducibility values but also, regarding the Cu-NPs treatments, they can be grouped in at least two major distinguishable discriminating groups: Group 1, which represents the control treatments (that is, without Cu-NPs), and Group 2, representing those treatments in which Cu-NPs were added to the culture media (PC2, at 0.5, 0.75 and 1 mg/mL, respectively). Regarding the sampling points (3 and 6 dai), despite the visible differences, they only explain a low percentage of the variance (PC1; Figure 4a). Based on these results, pairwise comparisons were performed to identify differentially expressed genes involved in Cu-NPs responses. Comparisons performed were 0.5, 0.75, and 1 mg/mL versus 0 mg/mL (control) at 3 and 6 dai, respectively. DEseq2 R package was used to calculate differential expression between these pairs of compared samples. In total, there were 5476 *F. kuroshium* genes with differential expression of two-fold or greater (Log_2_FC = ±1) and an adjusted significant *p* value of ≤ 0.05 at three dai (Table S7).

Conversely, the DEGs were slightly more abundant (6787) once six days after inoculation elapsed (Table S8). Venn diagram comparison of DEGs showed that a high percentage of DEG was shared at both sampling points analyzed (3 and 6 dai, respectively). There is a similar percentage of up- and downregulated genes (53.9% and 65.3%; Figure 4c). The DEGs resulted as higher as the concentrations of Cu-NPs increased (Figure 4 and Tables S7 and S8). These data suggest that even when colony morphology and mycelial growth inhibition are more significant at six dai, fungal molecular responses to overcome toxic stress and maintain cell viability are triggered at earlier stages and probably kept over time, while the stress is present and the fungal cells lose their viability.

Pairwise Pearson’s correlation coefficients (r) were estimated using the lists of DEGs to compare transcriptional responses (at global level) between the distinct Cu-NPs concentrations. That is, for each sampling point (3 and 6 dai), coefficients (r) were estimated between 0.5–0.75 mg/mL, 0.5–1 mg/mL, and 0.75–1 mg/mL. Student’s *t* test was used to assess whether correlations were significant (*t* test, *p* ≤ 0.05). The transcriptional responses seem to be similar based on these analyses. According to r values (ranging from 0.845 to 0.985), no significant differences exist between the distinct Cu-NPs concentrations or the sampling time points (Table S9). Similar to the transcriptional responses, colony morphology and mycelial radial growth inhibition percentages showed that the Cu-NPs at concentrations as low as 0.5 mg/mL have a comparable effect to those with higher concentrations (0.75 and 1 mg/mL). Transcriptional responses that may be involved are similar regardless of the time point analyzed, 3 or 6 dai.

### 3.4. Gene Ontology Enrichment Analysis of Cu-NPs Responsive Genes

**Figure 5:**
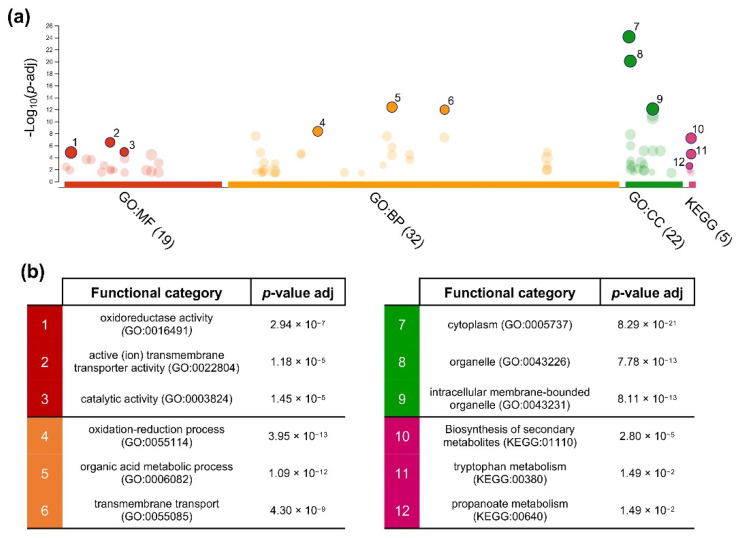
Enrichment of the GO terms and KEGG metabolic pathways by DEGs responsive to Cu-NPs. (**a**) Manhattan plot illustrating the significantly enriched (g: SCS threshold, *p* value ≤ 0.05) terms. The top three terms (solid colored dots) were numbered to distinguish them from the rest (dimmed colored dots). The name of each of these categories and its statistical significance are also shown (**b**).

To further examine the functions of the DEGs, an enrichment analysis of GO functional categories and KEGG metabolic pathways was performed using g: Profiler web server (see Methods for details). Nineteen molecular function (MF) terms, 32 biological processes (BP) terms, and 23 cellular components (CC) terms were significantly enriched by 4028 of the DEGs (66.4% of total), which were identified at both sampling points (Table S10). The top three GO terms enriched on each of these three major categories (Figure 5) included for MF were: oxidoreductase activity (GO:0016491), active (ion) transmembrane transporter activity (GO:0022804), and catalytic activity (GO:0003824); for BP: oxidation-reduction process (GO:0055114), organic acid metabolic process (GO:0006082), and transmembrane transport (GO:0055085); and for CC: cytoplasm (GO:0005737), organelle (GO:0043226), and intracellular membrane-bounded organelle (GO:0043231). Three KEGG metabolic pathways which were significantly enriched (*p* value ≥ 0.05) by DEGs were biosynthesis of secondary metabolites (KEGG:01110), tryptophan metabolism (KEGG:00380), and propanoate metabolism (KEGG:00640) (Figure 5 and Table S10).

### 3.5. Fusarium kuroshium Genes Involved in Transport, Homeostasis, and Cooper Toxicity and Resistance

There is still a limited understanding of the resistance mechanisms deployed by fungi to cope with the toxicity caused by Cu-NPs. Some of these molecular mechanisms have been studied mainly in yeast (Saccharomyces cerevisiae), but filamentous fungi reports are scarce. Downregulation of metal ion importers, utilization of metallothionein, metallothionein-like structures, and ion sequestration to the vacuole have been implicated in yeast’s resistance to metals (zinc, copper, iron, and silver, among others). In filamentous fungi, however, metal resistance relies heavily upon the export of these ions [41]. Therefore, we extensively searched genes involved in copper resistance using previous reports and recent reviews as a starting point [41,42,43,44,45,46]. *F. kuroshium* homologs of the genes from either yeast or filamentous fungi were identified on the lists of DEGs (Table S11). We found several homologs of enzymes involved in copper transport and homeostasis previously reported in yeasts, for example, some *F. kuroshium* genes homologs to FRE1 (FuKu07004) and FRE7 (Fu-Ku03123, FuKu04041, FuKu10175), both ferric/cupric-chelate reductases that, except for FuKu03123, were strongly downregulated (Log_2_FC values ranged from −3.32 to −10.17) in all Cu-NPs analyzed treatments (0.5, 0.75, and 1 mg/mL). FRE1 [47] (and other members of this gene family [46]) are metallo-reductases that reduce both cupric (Cu^2+^) and ferric (Fe^3+^) ions by bounding to two distinct transcription factors, MAC1, and ATF1, respectively [48,49,50]. No homologs to these transcription factors were identified in the *F. kuroshium* proteins coding genes set, suggesting that, perhaps in Fusarium species, distinct transcription factors are involved in a similar response. In addition to FRE proteins, homologs to low- (CRT2; FuKu05634) and high- (CRT3; FuKu05575, Fu-Ku07307) affinity copper transporters were also repressed or downregulated in all tested Cu-NPs treatments. Similar expression patterns (significant downregulated) were found for other homologs to copper transporters such as PIC2 (FuKu08121) and CCC2 (FuKu08773), proteins which shuttle Cu^+^ from the cytoplasm to the mitochondrial matrix and Golgi bodies, respectively [51,52,53].

Other enzymes such as ferroxidases 3 (FET3; FuKu00497, FuKu00629, FuKu01416, FuKu05480) and 5 (FET5; FuKu12927, FuKu08718) were significantly upregulated even when they were required for uptake and oxidation of ferrous iron. It is known that they require copper as a cofactor for properly functioning [54]. As expected, an ortholog to the *CrpA* gene from *Aspergillus fumigatus* (FuKu02881) was also significantly induced (Log_2_FC values > 8). This gene participates as a copper export and is an intermediate of copper’s reactive oxygen species responses [55].

Other groups of upregulated genes were those involved in the biosynthesis of cell wall components such as chitin (BioCyc ID: PWY-6981; enzymes: NTH1; FuKu06343, HXK2; FuKu07788 and FuKu11848, PCM1; FuKu08072, and QRI1; FuKu07424) and b-glucans (GO-term: fungal-type cell wall beta-glucan biosynthetic process (GO:0070880); genes: *Rot2*; *FuKu01065*, *FuKu04120*, *FuKu08774* and *FuKu09863*, *Cwh41*; *FuKu09879*, *KAR2*; *FuKu03662*, and *Kre5*; *FuKu01731*), besides those which participate in copper detoxification by Golgi-to-vacuole transport by the AP-3 adapter complex in the alkaline phosphatase pathway and in the carboxipeptidase Y pathway, which transport cargo to the vacuole through endosomal intermediates ([43] proteins: GDA1; FuKu09812, GYP1; FuKu05874 and FuKu06284, RUD3; FuKu02081, HOC1; FuKu04569, HOC1; FuKu11979, IMH1; FuKu00391, VPS25; FuKu03105, SNF7; FuKu06560, PEP1; FuKu07887, NHX1; FuKu09337, APS3; FuKu05521, CCC1; FuKu05129).

**Figure 6:**
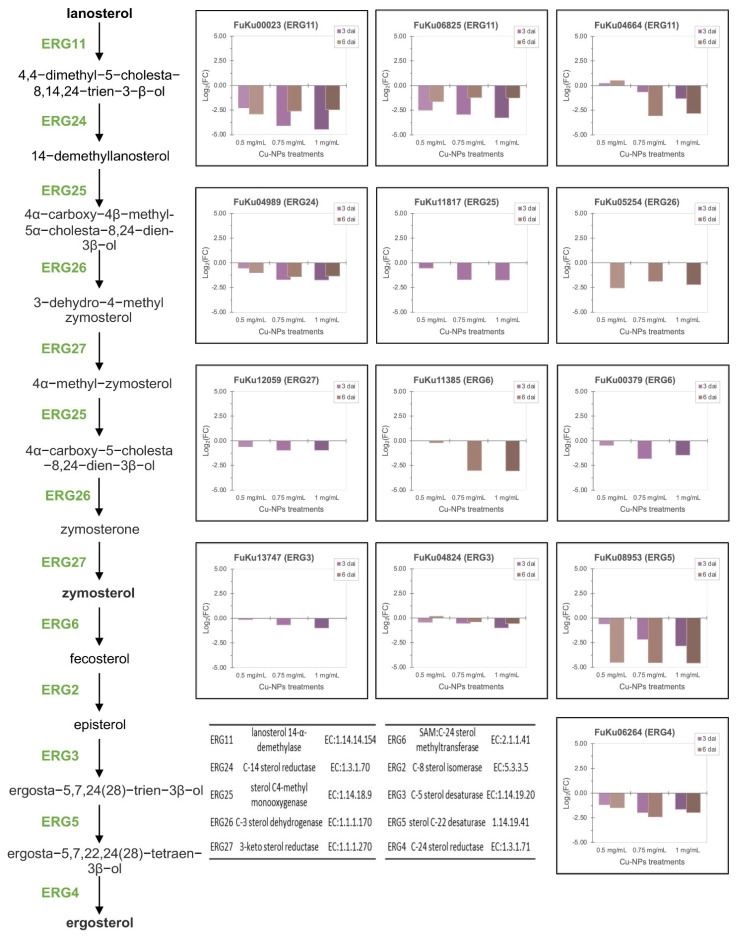
Ergosterol biosynthesis pathway (left) shows the expression profiles of *F. kuroshium* genes (ERG enzymes) involved. These expression profiles were significant in RNAseq differential expression analysis and are represented as Log_2_FC values. Enzyme names and corresponding Enzymatic Commission (EC) numbers are also shown.

Consistent with previous studies show that exposure of yeasts to trace amounts of metals such as copper, lead, iron, or zinc produce toxicity or death by interfering with several biological processes, including the ergosterol biosynthesis [41,56]. We found that *F. kuroshium* downregulated most of the genes involved in this biosynthetic pathway, even some of those represented in multi copies (paralogs) in *Fusarium* genomes in response to the majority of Cu-NPs concentrations (Figure 6 and Table S12). Similarly, Candida albican’s nine sterol-response elements (ERG1, ERG2, ERG5, ERG6, ERG10, ERG11, ERG24, ERG26, and ERG27) are regulated by UPC2 transcription factor [57,58]. In *F. kuroshium*, most of these enzymes (Figure 6) show downregulated patterns in response to the Cu-NPs treatments. While it is true, it has been proven that the efficiency of ergosterol biosynthesis is determined by some limiting enzymes, and more crucially by the optimal coordination of the regulation of encoding genes involved in this biosynthetic pathway [59]. In ascomycetes and basidiomycetes, there is a positive correlation between the synthesized metabolites (ergosterol and its precursors) and expression profiles of genes codifying for enzymes involved in its biosynthesis, mainly in those genes related to the post-squalene pathway [60,61].

## 4. Discussion

This study showed that Cu-NPs exhibit a better antifungal activity against *F. kuroshium* than cupric hydroxide. Some effects that were observed at concentrations ranging from 0.1 to 0.5 mg/mL were color changes of the fungal colony (Figure 1). This effect has been observed in other plant pathogenic fungi, such as *F. solani*, *Neofusicoccum sp*., and *F. oxysporum* [27]. In fungi, pigment production is related to melanin and carotenoid synthesis and is considered a defense mechanism against external stress [62]. In addition, it has been proven that the roles of fungal melanin include, among others, the scavenging of free radicals [63]. This is consistent with our results since the synthesis of pigments might be a mechanism by which *F. kuroshium* seeks to counteract the oxidative stress produced by Cu-NPs.

DEGs’ enrichment analyses of GO terms and KEGG metabolic pathways show that the top three enriched terms in the BP category defend against copper toxicity. Those processes are related to each other and correspond to oxidation–reduction processes, organic acid biosynthesis, and the active transport of ions through the plasma membrane and the membranes that bound the organelles.

Organic acid production has been suggested to give a competitive advantage to filamentous fungi over other organisms by decreasing the pH and impacting metal detoxification [64,65]. The decrease in pH upon their secretion may give a competitive advantage to the acid-tolerant filamentous fungi, depending on the environment in which they grow [66]. For saprophytic and wood-decaying fungi, pH acidification, caused by oxalic acid production (another significantly enriched GO term; GO:0043436), leads to acid-catalyzed hydrolysis of holocellulose [67,68,69]. Depending on their concentration, type of metal, and pH, organic acids can also be complex with di- and tri-valent metals (Fe, Cu, Al, among others), explaining their essential role in metal detoxification [65]. The degree of complexation is also dependent on the organic acid involved (number and proximity of carboxyl groups). This result suggests that *F. kuroshium*, at least in part, seeks to counteract the toxicity caused by Cu-NPs by synthesizing some organic acids.

Fungal–copper interactions are necessary for the activation of metalloproteins involved in biochemical processes. This includes the activation of superoxide dismutase, which is responsible for cellular detoxification of reactive oxygen species (ROS) and activation of cytochrome c oxidate, a catalyst within the electron transport chain [41]. Copper [56], zinc [70], and silver [71,72] NPs interfere with ergosterol biosynthesis, increasing leakage of the cytoplasmic contents, depolarization, occurrence of ROS, and reducing cell wall integrity in yeasts. This explains the significant enrichment of the oxidation–reduction processes (GO:0055114) and enzymes with oxidoreductase activity (GO:0016491. In addition, metallothioneins (proteins that use metal ions as cofactors that possess a cysteine-rich domain) bind free cytosolic ions as a mechanism of ion storage or detoxification. In metal-deficient conditions, ions may be released back into the cellular environment [73]. Specific protein intracellular transporters are involved in this movement of ions to organelles either for storage or as cofactors for protein functioning [41,53] It is known that interference with these systems causes a homeostatic imbalance, resulting in toxicity [41].

Regarding KEGG terms, we consider that the secondary metabolites pathway (KEGG:01110) could be significantly enriched due to the pigments produced by *F. kuroshium* (Figure 1). Meanwhile, the enrichment of the tryptophan biosynthetic pathway (KEGG:00380) is consistent with Jo et al., who in 2017 [43], used microarrays and deletion mutants to identify genes in *Saccharomyces cerevisiae* involved in the toxic response against iron and copper. In that study, the changes in the expression of genes in the tryptophan biosynthesis pathway were specific to the copper response, suggesting that at least in yeasts, the mechanisms to deal with high concentrations of these two metals are specific for each of them. The role of the tryptophan biosynthetic pathway in the overload of copper in yeasts and some fungi such as *F. kuroshium* is still unknown. However, it has been suggested that its involvement is associated with the metabolites produced during degradation in the kynurenine pathway, which have antioxidant properties [74], or its radical-scavenging activity, as superoxide radicals are used as a cofactor to cleave the pyrrole ring in tryptophan [75]. Alternatively, it is also possible that tryptophan may be required as a critical residue in specific proteins involved in the defense against copper toxicity [43].

**Figure 7:**
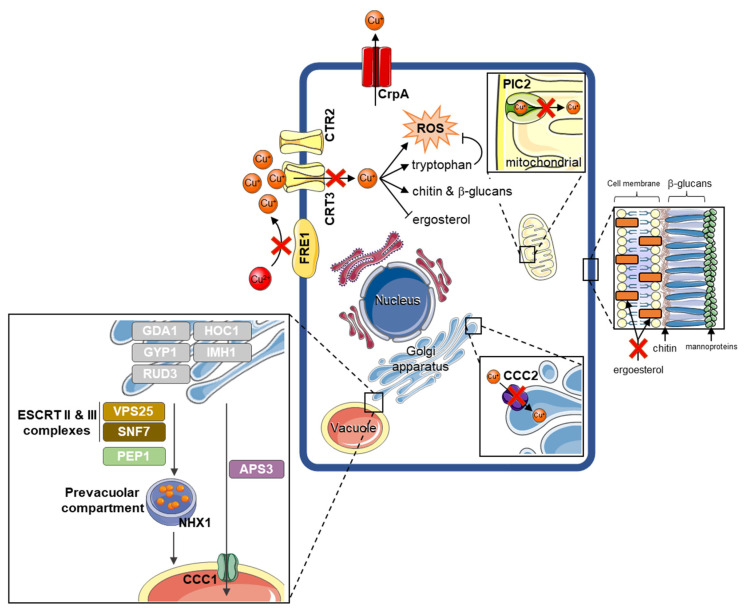
Schematic representation of mechanism involved in detoxification and the resistance to Cu-NPs treatments in *F. kuroshium* species. The membrane transporters and other proteins represented in the cell are named based on its yeast *(Saccharomyces cerevisiae*) homologs.

Based on the expression profile of some DEGs, our data suggest that *F. kuroshium* counteracts the toxicity caused by Cu-NPs through several mechanisms as shown in Figure 7, including a significant decrease in the transcription of genes codifying both the reductase that reduces extracellular copper (Cu^2+^), and the low- and high-affinity membrane transporters that shuttle the reduced copper (Cu^+^) to the cytoplasm. In addition, several transporters in intracellular membrane-bounded organelles are also downregulated. These results suggest that *F. kuroshium* tries to considerably reduce the shuttle of Cu^+^ to some organelles as Golgi bodies and the mitochondria. In contrast, in toxic copper concentrations, the overexpression of the CrpA transporter may occur as a defense mechanism to prolong its life by exporting Cu^+^ from the cytoplasm to the extracellular space. The overexpression of some metalloproteins and other proteins that use copper ions as cofactors (e.g., ferroxidases) can also be considered as copper storage or a detoxification mechanism because these proteins bind free cytosolic ions, releasing them back into the cellular environment in metal-deficient conditions [41,73].

Considering the expression profile (upregulated) of several genes whose coding proteins form the endosomal sorting complex are required for transport (ESCRT), we suggest that both *F. kuroshium* such as *S. cerevisiae* (and probably another eucaryotic organism), employ this detoxification pathway in response to the copper overload [43]. No DEGs were found for the retromer complex; this suggests that intracellular traffic of copper ions (or proteins that bind it) may occur preferably in one way (from Golgi to vacuole). In addition, high levels of Aps3 suggest that the AP-3 complex (which, similar to ESCRT, also converges toward the vacuole) is also involved in copper detoxification. It has been reported that even when yeast molecular responses to iron and copper share some mechanisms, the AP-3 adapter complex in the alkaline phosphatase pathway is mainly involved in iron overload resistance [41,43].

Particular concentrations of copper cytosolic ions also interfere with the redox balance and increase the generation of reactive oxygen species [76]. High amounts of reactive oxygen species (ROS) can induce autophagy, apoptosis, and cell death [77]. Other consequences of free cytosolic Cu^+^ ions reduce ergosterol biosynthesis and increase tryptophan synthesis. As mentioned above, it has been discussed that the participation of tryptophan in the response to copper-induced toxicity could be through antioxidant properties of the metabolites produced during degradation in the kynurenine pathway, which has radical-scavenging activity as a superoxide radical (a radical that contributes to oxidative stress) [43,74,75]. The reduction in ergosterol biosynthesis decreases cell wall integrity, increases cellular leakage and depolarization, and increases the occurrence of ROS [70]. We found that the genes involved in chitin and b-glucans biosynthesis are upregulated. This suggests that maybe *F. kuroshium*, faced with a constant block in the synthesis of ergosterol, seeks to maintain the cell wall integrity by increasing the production of its other primary components (e.g., chitin and β-glucans).

SEM micrographs (Figure 3) show a loss in cell wall integrity. Our analyses discussed before can explain this phenomenon by observed changes in the transcript levels of the genes involved in ergosterol biosynthesis. However, SEM micrographs also revealed that macroconidia, such as hyphae, were severely damaged and can only be found at concentrations as low as 0.1 mg/mL of Cu-NPs. This suggests that concentrations slightly higher (≥0.25 mg/mL) not only inhibit *F. kuroshium* growth but also interfere in the formation of asexual spores such as macroconidia. We cannot explain this observation in light of the generated results; however, this effect of Cu-NPs treatments will be addressed in future works. Together all these results suggest that the toxicity of Cu-NPs affects several biological processes that compromise cell viability.

## 5. Conclusions

The presented work proves that using Cu-NPs could be considered as a highly efficient alternative with better antifungal properties than other formulations commonly proposed and commercially available fungicides such as cupric hydroxide. Molecular responses to Cu-NPs treatments analyzed by RNA-seq suggest that *F. kurhosium* counteracts the toxicity caused by free cytosolic copper ions through different mechanisms. These mechanisms include avoiding copper reduction, internalization, and intracellular movement. For this purpose, the amount of high- and low-affinity transporters and other specific transporters decreases considerably. In addition, free copper cytosolic ions also decrease by binding to copper-dependent proteins, which are strongly induced, including metallothionein. The overexpression of other transporters exporting Cu^+^ from the cytoplasm to the extracellular space is also essential in the detoxification process. These detoxification mechanisms seek to maintain cell viability, which is ultimately compromised due to the loss of cell wall integrity resulting from reduced ergosterol synthesis. Cytosolic leakage and depolarization increase the occurrence of ROS, which induces autophagy, apoptosis, and cell death.

## Data Availability

The RNA-seq data were deposited in the Short Read Archive (SRA) of the National Center for Biotechnology Information (NCBI) with accession number PRJNA805244.

## Supplementary Materials

The following are available online at https://www.mdpi.com/article/10.3390/jof8040347/s1, Table S1: Summary of Illumina sequencing, Table S2: Annotation of the Fusarium kuroshium genes by homology-based inference, Table S3: Gene Ontology-based functional characterization of the Fusarium kuroshium genes, Table S4: KOG terms inherited to Fusarium kuroshium genes, Table S5: KEGG pathways inherited to Fusarium kuroshium genes, Table S6: Expression profile matrix of Fusarium kuroshium genes, Table S7: Fusarium kuroshium differentially expressed genes (DEGs) at 3 dai, Table S8: Fusarium kuroshium differentially expressed genes (DEGs) at 6 dai, Table S9: Pearson’s correlation matrix, Table S10: Over-represented GO terms on the list of Fusarium kuroshium DEGs (Gene Ontology enrichment analysis), Table S11: Common copper-responsive genes shared between yeast and Fusarium kuroshium, Table S12: Fusarium kuroshium differentially expressed genes (DEGs) involved in ergosterol biosynthesis.
